# Invasive Seaweed *Rugulopteryx okamurae*: A Potential Source of Bioactive Compounds with Antioxidant Activity

**DOI:** 10.3390/antiox13111298

**Published:** 2024-10-25

**Authors:** Radia N. A. Belhadj, Cristina Mellinas, Alfonso Jiménez, César Bordehore, Maria Carmen Garrigós

**Affiliations:** 1 Department of Analytical Chemistry, Nutrition & Food Sciences, University of Alicante, San Vicente del Raspeig, 03690 Alicante, Spain; rnab1@alu.ua.es (R.N.A.B.); cristina.mellinas@ua.es (C.M.); alfjimenez@ua.es (A.J.); 2 Department of Ecology, University of Alicante, 03690 Alicante, Spain; cesar.bordehore@ua.es; 3 Multidisciplinary Institute for Environmental Studies “Ramon Margalef”, University of Alicante, 03690 Alicante, Spain

**Keywords:** *Rugulopteryx okamurae*, invasive seaweed, bioactive compounds, antioxidant activity, valorization

## Abstract

*Rugulopteryx okamurae* (RO) is a species of brown seaweed that has invaded several shorelines worldwide, including the Spanish Mediterranean and the Strait of Gibraltar coasts, causing serious environmental and economic problems. This work aimed to characterize the bioactive composition of RO. A high content of carbohydrates (58.7 ± 2.6 wt%), fats (17.1 ± 0.4 wt%), and ashes (14.3 ± 0.2 wt%) were found, together with lower protein content (5.5 ± 1.8 wt%). Holocellulose was the most abundant polysaccharide fraction (49.2 ± 1.3 wt%), showing 43.4 ± 2.0 wt% of cellulose and 5.8 ± 0.7 wt% of hemicellulose, followed by lignin (18.9 ± 2.5 wt%). The monosaccharides composition showed a high level of glucose (13.2 ± 1 wt%) and glucuronic acid (9.3 ± 0.5 wt%). RO contained high levels of essential nutrients (Ca, K, Na, S, Mg), trace minerals (Mn, Mo, Se, and Cu), and some toxic heavy metals (Ni, Cd, As). The main fatty acid present in RO was palmitic acid (C16:0, 30.8 ± 3.0 mg/100 g), followed by myristic acid (C14:0, 19.3 ± 2.4 mg/100 g) and eicosatetraenoic acid (C20:4, 19.2 ± 1.3 mg/100 g). The extract obtained by microwave-assisted extraction (MAE) presented significant contents of polyphenols (2.7 ± 0.2 mg GAE/g) and antioxidant activity (3.0 ± 0.4 mg TE/g DPPH, 4.5 ± 0.3 mg TE/g ABTS, 4.7 ± 0.3 mg TE/g FRAP). Six main polyphenols were identified by HPLC-MS/MS, showing higher contents of gallic acid (20.7 ± 1.5 mg/g) and chlorogenic acid (9.7 ± 0.5 mg/g). These results highlight the possibilities offered in the valorization of RO to obtain bioactive compounds with antioxidant performance in several applications.

## 1. Introduction

Seaweeds or macroalgae are multicellular organisms living in the sea and oceans and are able to perform photosynthesis reactions [1], showing different shapes, sizes, colors, and compositions [2]. *Rugulopteryx okamurae* (RO) is a brown alga, which belongs to the *Dictyotaceae* family. It is also known as *Dilophus marginatus, Dictyota marginata*, or *Dictyota okamurae*, and it is native to the northwestern Pacific Ocean, covering major coastal areas in Japan, China, Taiwan, Korea, and the Philippines [3]. RO dwells on sublittoral rocky substrata from 0.5 to 35.0 m deep, but it was reported to be present up to 40.0 m deep in the Ceuta Bay [4]. In the Mediterranean, it is an invasive seaweed first detected in 2002 in the Thau lagoon of the French coast, being accidentally introduced along with Japanese oysters. The first reports on the presence of RO in Spanish seas date from 2015, when it was detected in the coasts of Ceuta (North Africa). Just one year later, RO covered many illuminated rocky bottoms seabed in this area, resulting in more than 5000 tons of algal biomass removed from Ceuta’s beaches [5], reaching more than 90% coverage in some areas [6]. The growing of RO in the Spanish Mediterranean coasts and, in particular, those in the surroundings of the Strait of Gibraltar is highly favored, with a fast expansion and causing serious environmental and economic problems. In 2020, more than 3 million square meters of the seabed in the Gibraltar Strait Natural Park was already invaded by RO at different depths [3]. This macroalga was recently included in the Spanish catalog of invasive alien species (order TED/1126/2020 of the Ministry of Ecological Transition) [7]. Over the past two years, RO has spread very aggressively, resulting in the accumulation of thousands of tons of upwelling seaweed [6]. Invasive marine species, and particularly RO, have largely contributed to the collapse of fisheries and aquaculture and major losses in touristic and marine infrastructures by modifying the invaded habitat, displacing native species, and altering food webs [8]. Invasive algae have been considered as priority pest species with significant negative ecological and socio-economic effects [9].

Seaweeds can be used as human food or as raw materials to obtain different valuable natural products of nutritional interest, although this strategy is not fully commercially exploited yet. Some algae species, belonging to the *Halophila*, *Zostera*, *Syringodium Posidonia*, and *Thalassia* genus, were recently investigated [10]; although, currently, approximately 32.4 million tons of algae are produced with a financial value of USD 13.3 billion per year, just 2.9 million tons of them represent wild seaweeds [11]. At least 386 seaweed species were identified, including 214 rhodophytes, 116 chlorophytes, and 56 phaeophytes [12]. There has been some recent and increasing interest in seaweed varieties due to their content in numerous bioactive compounds with potential use in several fields, including food, pharmaceuticals, and medicine [13]. It is known that seaweeds are rich in compounds and active principles important for human health, including dietary fiber (soluble and insoluble), polysaccharides, carotenoids, minerals, vitamins, and other macromolecules, such as carbohydrates, proteins, essential fatty acids, lipids, amino acids, and polyphenols [14]. Many factors are relevant in seaweed’s composition, particularly their chemical structure and quantities depending on their taxonomy and their geographic and environmental distribution [15]. Algae belonging to the *Dictyotaceae* family are an important source for almost 40% of the metabolites isolated from brown algae [16]. These compounds proved to have different biological activities (antioxidant, antibacterial, antifungal, antitumor, antiviral, anti-inflammatory, neuroprotective), with a high potential to act as functional agents in innovative applications in the cosmeceutical, food, and pharmaceutical industries [17]. The rich bioactive composition of seaweeds is related to their ability to survive in complex environments, generating high quantities of secondary metabolites that are not produced in terrestrial plants [18]. Seaweeds have also gained attention as alternative renewable sources for “third-generation” biofuel production [17] and as green adsorbents in the bioremediation of polluted seawater due to their ability to capture metal contaminants from the environment [19].

Although some studies have highlighted the potential of RO collected from Spanish Andalusian beaches as a valuable source of bioactive compounds [20], there is still just a few reports on its chemical composition, in particular, on the polyphenolics profile and antioxidant properties of this species. In addition, it should be considered that different chemical compositions can be expected depending on RO’s geographical location as a result of macroalga adaptation to different marine environments (temperatures and currents). Thus, this work deeply examines, for the first time, the bioactive composition and antioxidant activity of RO collected from the Gibraltar area as a new sustainable source of bioactive compounds in different industrial sectors. This study offers valuable new insights of valorization approaches to reduce the environmental and economic problems of RO waste accumulation by creating new functional added-value products. The proximate composition, polysaccharides, minerals content by inductively coupled plasma-mass spectrometry (ICP-MS), monosaccharides content by high-performance anion-exchange chromatography with pulsed amperometric detection (HPAE-PAD), fatty acids methyl esters (FAMEs) profile by gas chromatography-mass spectrometry (GC-MS), and thermal stability (thermogravimetric analysis, TGA) were determined. The total phenolics content (TPC), antioxidant activity by three spectrophotometric methods, and phenolics profile by high performance liquid chromatography-mass spectrometry-mass spectrometry (HPLC-MS/MS) of extracts obtained by microwave-assisted extraction (MAE) were also analyzed, and the correlation between phenolics and antioxidant properties was assessed.

## 2. Materials and Methods

### 2.1. Chemicals, Sample Collection and Preparation

**Figure 1:**
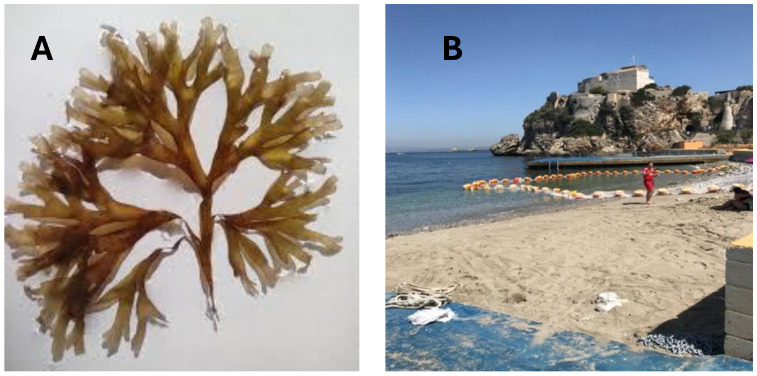
Brown seaweed RO (**A**) and sampling location in Gibraltar (**B**).

All analytical-grade standards and chemical reagents were purchased from Sigma-Aldrich, Inc. (St. Louis, MO, USA). The brown seaweed RO was collected from Camp Bay beach (36°7′9.3684″, −5°21′5.1984″), Gibraltar, in May (size around 1–10 cm, Figure 1). Sea temperature and salinity values were 16 °C and 36.5 PSU, respectively. Raw and fresh seaweeds impurities were removed by rinsing with water, and they were further cut into small pieces. Samples were placed in an ultra-freezer for 2 h and lyophilized (Telstar, LYOQUEST-55 PLUS, Terrassa, Spain) for 4–5 days. Dried samples were ground to a fine and homogeneous powder by using a ZM 200 high-speed rotatory mill (Restch, Hann, Germany) with a 0.5 mm pore size sieve and stored in hermetic bags at room temperature in the dark. All analyses were carried out in triplicate.

### 2.2. Proximate Composition

Moisture content was determined according to TAPPIT264 cm-97 standard [21] heating samples in an oven at 105 °C until constant weight. The ash content was determined following TAPPI T211 om-02 [22] standard by the gravimetric method in a muffle furnace at 525 °C. Solvent extractives were determined by solid–liquid extraction (Soxhlet) using ethanol–toluene (1:2, *v*/*v*) for 6 h according to TAPPI T204 cm-97 standard [23]. The fat content was assessed according to AOAC 920.85 [24] method by Soxhlet extraction using petroleum ether for 6 h. Protein content was determined using the Kjeldahl method, considering a protein conversion factor of 6.25 [25]. Carbohydrates content was calculated by difference from 100% of the percentile contents of all other major components (moisture, ash, fat, and proteins). Lignin contents were determined following TAPPI um 250 (acid soluble) [26] and TAPPI T222 om-02 (acid insoluble) [27] methods. Holocellulose and α-cellulose contents were determined according to Wise et al. [28] and Rowell [29], respectively. Hemicelluloses were calculated by the difference in holocellulose and α-cellulose contents. Results were expressed in g/100 g dry RO (wt%).

### 2.3. Fatty Acids Profile

The fatty acids composition of the fat fraction extracted from RO was determined by GC-MS. Fatty acid methyl esters (FAMEs) were obtained by following the ISO 12966-2:2017 [30] with an Agilent 5973 gas chromatograph-MS (Agilent Technologies, Santa Clara, CA, USA) using a BPX70 capillary column (60 m × 0.25 mm × 0.25 μm), and helium was used as carrier gas (1 mL/min). The column temperature was initially set at 120 °C for 3 min followed by an increase up to 245 °C at 3 °C/min (hold for 10 min). The injector and detector temperatures were set at 250 °C and transfer line was kept at 300 °C for the whole run. Samples were injected (1 μL) with 1:20 split ratio. Mass spectra were obtained in the scanning mode (30–450 *m*/*z*) and results were compared to the National Institute of Standards and Technology (NIST) database and analytical standards for FAMEs identification. The quantitative analysis of FAMEs was performed using external calibration (5–600 mg/kg, R^2^ = 0.9960). A Supelco 37 Component FAMEs standard mix (Sigma-Aldrich, St. Louis, MO, USA) was used. Results were expressed as mg FAMEs/g dry RO.

### 2.4. Monosaccharides Content

Monosaccharides were determined following the method reported by Shi et al. [31]. An amount of 2 mg of moisture-free sample was diluted in 250 µL of H_2_SO_4_ (72 wt%) and hydrolyzed at ambient temperature for 2 h. Then, 2.75 mL of distilled water was added and the whole mixture was left at 100 °C for 3 h. After cooling to room temperature, sample was filtered, and 1.5 mL of the supernatant was diluted to 10 mL with MQ water. A Metrohm 850 ProfIC AnCat-MCS ion chromatograph (Metrohm AG, Herisau, Switzerland) with pulsed amperometric detection (HPAE-PAD) was used equipped with a Metrosep Carb 2 column (250 cm × 4 mm). The mobile phase was composed of solvent A (1 mM NaOH/1 mM sodium acetate) and solvent B (300 mM NaOH/500 mM sodium acetate). The following gradient elution program used was: 0 min, 100% A; 30 min, 100% A; 38 min, 50% A; 46 min, 50% A; 52 min, 100% A. An amount of 10 μL of sample was injected at a flow rate of 0.6 mL/min at 35 °C. Different monosaccharide standards were used for quantification (Sigma-Aldrich, St. Louis, MO, USA) and results were expressed in g/100 g dry RO (wt%).

### 2.5. Minerals Content

Mineral elements were determined according to Cofrades et al. [32]. An amount of 0.2 g of ash was digested in 4 mL of concentrated HNO_3_ for 48 h at 25 °C. Then, 6 mL of Milli-Q water were added, and the resulting solution was used for minerals determination using an ICP-MS triple quadrupole Agilent model 8900 (Agilent technologies, Santa Clara, CA, USA). All analyzed minerals were calculated based on external calibration and the results were expressed in mg/100 g dry RO.

### 2.6. Thermal Stability

Thermogravimetric analysis (TGA) of the moisture-free sample was performed using a TGA/SDTA 851 Mettler Toledo thermal analyzer (Schwarzenbach, Switzerland). An amount of 6 mg of sample was heated from 25 to 900 °C at 10 °C/min under nitrogen atmosphere (flow rate 50 mL/min).

### 2.7. Bioactive Compounds

#### 2.7.1. Microwave-Assisted Extraction (MAE)

MAE was performed with a FLEXIWAVE™ microwave oven (Milestone Srl, Bergamo, Italy), according to the method of Čagalj et al. [33]. Samples (3.5 g) were added into a round-bottom flask with 70 mL of EtOH 70 wt%. Sample was introduced in the microwave oven for 34 min at 60 °C and 500 W. The solution was further centrifuged at room temperature (5000 rpm, 8 min) and the supernatant was recovered. The solvent was eliminated with a rotary evaporator and then, the extract was lyophilized (Telstar, LYOQUEST-55 PLUS, Terrassa, Spain). The extraction yield was calculated using Equation (1).

Yield (%) = (weight of dried RO extract)/(weight of dried RO used for extraction) × 100(1)

#### 2.7.2. Antioxidant Activity

Antioxidant activity was assessed by using three spectrophotometric methods, namely the 2,2-diphenyl-1-picrylhydrazyl (DPPH), 2,2-azino-bis-ethylbenzothiazoline-6-sulphonic acid (ABTS) and ferric reducing antioxidant power (FRAP) assays according to the method reported by Solaberrieta et al. [34], using a Biomate-3 UV/Vis spectrophotometer (Thermo Spectronic, Mobile, Waltham, MA, USA). An amount of 20 mg of ethanolic extract were previously dissolved in 10 mL of EtOH 70 wt%. All results were expressed as mg Trolox equivalents/g dry RO. For DPPH radical scavenging activity, 0.1 mL of RO solution was mixed with 2 mL of freshly prepared DPPH solution. Samples were incubated in the dark for 2 h and the absorbance was measured at 517 nm. The scavenging activity (%) was calculated as percentage of inhibition (Equation (2)). The inhibition of DPPH free radicals was calculated by comparison with a Trolox calibration curve (10–250 mg/kg, R^2^ = 0.9918).

Antioxidant activity (%) = [(A_Control_ − A_Sample_)/A_Control_] × 100 (2)

where A_Control_ and A_Sample_ are the absorbance values of control (DPPH or ABTS solution without sample) and sample (DPPH or ABTS solution and sample), respectively.

ABTS radical scavenging activity was calculated by using 0.1 mL of sample mixed with 3 mL of the ABTS working solution. The mixture was further incubated in the dark for 2 h. The absorbance was measured at 734 nm and ABTS inhibition was calculated with a Trolox calibration curve (10–200 mg/kg, R^2^ = 0.9995). For the FRAP assay, 1 mL of sample was mixed with 3 mL of the freshly prepared FRAP reagent. This mixture was incubated in the dark for 1 h. The absorbance was measured at 593 nm and Trolox was used as standard for the calibration curve (10–250 mg/kg, R^2^ = 0.9985). The scavenging activity (%) was calculated as percentage of inhibition (Equation (2)).

#### 2.7.3. Total Phenolics Content (TPC)

TPC was determined by following the Folin–Ciocalteu assay as reported elsewhere [33]. An amount of 20 mg of ethanolic extract was dissolved in 10 mL of EtOH 70 wt%. Then, 0.5 mL of solution were mixed with 2.5 mL of Folin–Ciocalteu fresh reagent in distilled water (1:10, *v*/*v*) and 2 mL of Na_2_CO_3_ 7.5 wt%. After incubation for 30 min in darkness at 45 °C, the absorbance at 765 nm was assessed, with gallic acid as the calibration curve standard (25–130 mg/kg, R^2^ = 0.9991). Results were expressed as mg gallic acid/g dry RO.

#### 2.7.4. Phenolics Profile

The identification and determination of the main phenolics was performed using a high-performance liquid chromatograph coupled to mass spectrometry (HPLC-MS) 6490 Triple Quadrupole (Agilent technologies, Santa Clara, CA, USA) with an electrospray ionization (ESI) source. A Halo C18 (2.7 µm, 4.6 × 100 mm) column was used, injecting 5 μL at 1.0 mL/min flow rate. The mobile phase was composed of solvent A (Mili Q Water and 0.1 wt% formic acid) and solvent B (acetonitrile and 0.1 wt% formic acid). The gradient elution program used was as follows: 95% A (1 min), 65% A (12 min), 55% A (18 min), 25% A (28 min), 95% A (40 min). ESI conditions were selected as follows: gas temperature: 275 °C, gas flow: 11 L/min; sheath gas temperature: 300 °C, sheath gas flow: 12 L/min; nebulizer pressure: 40 psi; capillary voltage: negative 2800 V, positive 3500 V and V charging negative 1500. Multiple reaction monitoring (MRM) scan type was recorded in positive and negative ionization mode over the *m*/*z* 50–1000 D range. The characteristic MS fragmentation for each compound was analyzed, and calibration curves of target phenolic standards were obtained. Agilent Mass Hunter workstation B.07.01 software was used for data acquisition and quantitative analysis. Results were expressed in mg/g dry RO.

### 2.8. Statistical Analysis

All experiments were performed in triplicate and results were expressed as mean values ± standard deviation (SD) on a dry weight (dw) basis. Differences between values were based on confidence intervals by using Tukey’s test (*p* ≤ 0.05 significance level). Spearman’s test was used to determine correlations between the obtained data.

## 3. Results and Discussion

### 3.1. Proximate Composition

The chemical composition of seaweeds (proteins, polysaccharides, minerals, pigments, polyphenols, and lipid contents) can vary significantly depending on several factors, such as species and genera, harvesting period, and habitat conditions (water temperature, light, salinity, nutrients) [35]. The proximate composition of RO obtained in this work and those found in other studies are shown in Table 1. Moisture content in seaweeds usually ranges from 9 to 36 wt% [36]. RO showed a minor content of 4.4 ± 0.2 wt%, in agreement with results reported by Patel et al. [37] for red seaweed *Gracilaria corticata,* green seaweed *Ulva reticulata*, and brown seaweed *Sargassum cinctum*, with moisture contents ranging from 2.75 ± 0.01 to 7.90 ± 0.03 wt%. In general, the typical moisture content of brown seaweeds was reported to be below 32 wt% [38]. Ferreira-Anta et al. [20] found a higher moisture content of 8.06 ± 0.16 wt% for RO. Moisture content can be affected by drying conditions (time and temperature), such as decreasing water content with increasing drying time [39]. The low moisture value obtained in this study was related to the lyophilization process used, which was shown as an effective drying method for reducing moisture in seaweeds [40], also slowing down microbial growth and increasing the sample’s shelf-life during storage [38].

The ash content found in seaweeds ranged between 8 and 40 wt% [41]. Generally, seaweeds present high concentrations of crude ash, mainly due to their accumulation trend in minerals and heavy metals from seawater [35]. This behavior is related to the role of physodes, commonly found in surface cells, acting as a first filter to avoid heavy metals gaining access to the inner parts of the thallus [42], allowing the alga to grow in potentially toxic environments [43]. An ash content of 14.3 ± 0.2 wt% was obtained in RO. Lower values of 11.30 ± 0.08 wt% and 11.56 ± 0.68 wt% were found by Cebrián-Lloret et al. [44] and Ferreira-Anta et al. [20], respectively, for RO. In another study, Agustín et al. [45] found a higher ash content of 15.15 ± 0.37 wt% in RO. Considering other brown seaweeds, similar values were reported in *Gelidium pristoidesa* (14 wt%) and *Sargassum vulgaref* (14.2 wt%) [46]. Ash contents in seaweeds are generally higher compared to terrestrial vegetables other than spinach, varying between different species, geographical locations, and seasons [47]. Brown seaweeds have shown higher ash contents compared to other seaweed types [48], depending on the seawater environment and heavy metals uptake [49]. The high ash content obtained in RO can be directly related to a high content in mineral elements present in this macroalga.

**Table 1:** Proximate composition of RO (mean ± SD, n = 3).

Proximate Composition	This Study (g/100 g dw)	Other Studies (g/100 g dw)
Moisture	4.4 ± 0.2	8.06 ± 0.16 [20]
Ashes	14.3 ± 0.2	11.56 ± 0.68 [20] 11.30 ± 0.08 [44] 15.15 ± 0.37 [45]
Proteins	5.5 ± 1.8	16.43 ± 0.70 [20] 12.2 ± 0.2 [44] 49.05 ± 1.36 [45]
Fats	17.1 ± 0.4	6.17 ± 0.15 [20] 17.3 ± 3.2 [44] 4.02 ± 0.29 [45]
Carbohydrates ^1^	58.7 ± 2.6	60.4 ± 5.1 [44]
Polysaccharides		
Insoluble lignin	18.2 ± 2.4	4.49 ± 0.60 [45]
Soluble lignin	0.7 ± 0.1	-
Holocellulose	49.2 ± 1.3	-
α-cellulose	43.4 ± 2.0	13.6 [45]
Hemicelluloses	5.8 ± 0.7	-

^1^ Carbohydrates were calculated by difference = 100 − sum (moisture + ashes+ proteins + fat contents.

Proteins are present in algae in several forms and cellular locations, as components of the cell wall, enzymes, or bound to pigments and carbohydrates [50]. The protein content of brown seaweeds is generally low, with values ranging between 5 and 15 wt%, compared to green and red seaweeds (10–30 wt%) [51]. The protein content found in RO was 5.5 ± 1.8 wt%. Similarly, Laurie Eve Rioux et al. reported a total protein content of 5.0 ± 0.2 wt% for brown seaweed *Saccharina longicruris* [52]. In other studies, Yang et al. [53] and Abou-El-Wafa et al. [54] found a lower protein content for brown seaweeds *Padina gymnospora* (0.57 wt%) and *Sargassum subrepandum* (3.2 ± 0.12 wt%), respectively. Similarly, Winarni et al. [38] reported a lower protein content for brown algae *Sargassum* sp. and red algae *Eucheuma cottonii* with values of 3.048 wt% and 2.056 wt%, respectively. However, Cebrián-Lloret et al. [44], Ferreira-Anta et al. [20], and Agustín et al. [45] found a higher protein content for RO with values of 12.2 ± 0.2 wt%, 16.43 ± 0.70 wt%, and 49.05 ± 1.36 wt%, respectively. Variations in protein content can be related to several factors, such as seasonal and temperature fluctuations; proteins consumption in reproduction and growth; differences in species, geographical locations, and environmental conditions surrounding seaweeds [55]; and thallus maturation [56].

The fat content present in seaweeds is generally lower than 5 wt%, with brown seaweeds (*Phaeophyceae*) showing a higher content, followed by green (*Chlorophyta*) and red (*Rhodophyta*) seaweeds [57]. It was reported that some brown seaweed species, such as *Dictyotales*, can have a fat content up to 20 wt% [38]. A fat content of 17.1 ± 0.4 wt% was found for RO, belonging to the order *Dictyotales*, which could explain the relatively high value obtained. This result also agreed with the findings reported by El-Shenody et al. [58], obtaining a higher lipid content for brown algae *Dictyota dichotoma* (order *Dictyotales*) compared to brown algae *Turbinaria decurrens* (order *Fucales*). Similarly, Winarni et al. [38] found a lower fat content of 0.652 wt% in red seaweed, *Eucheuma cottonii*, compared to brown seaweeds, which can produce 3–4 times more fatty acids [59]. The obtained results are also in agreement with a previous study in brown seaweed RO, reporting a fat content of 17.3 ± 3.2 wt% [44], although lower contents were found by Agustín et al. [45] and Ferreira-Anta et al. [20] (4.02 ± 0.29 wt% and 6.17 ± 0.15 wt%, respectively). The fat content in macroalgae can be directly affected by the season and period of sample collection. The highest fat content for *Stypopodium schimperi* (2.16 wt%) in summer was reported compared to *Spyridia filamentosa* (0.08 wt%) in spring [25]. The fatty acid content could vary depending on environmental conditions to maintain the integrity of the cell membrane [60]. Moreover, different extraction conditions and solvent polarity may also influence fat recovery [61]. Seaweed lipids were reported as potential phytochemicals with bioactive properties, such as antibacterial, anticancer, antioxidant, and inflammatory, suggesting possible uses in nutraceutical, cosmeceutical, and pharmaceutical industries [62].

Carbohydrates are the most important components of metabolism, mainly providing energy for respiration and other metabolic processes [63]. Carbohydrates were the predominant fraction of RO (58.7 ± 2.6 wt%), in agreement with previous studies reporting contents in brown seaweeds ranging between 40−60 wt% [64]. Similar contents were found by Rioux et al. [65] and Cebrián-Lloret et al. [44] for brown seaweeds *Saccharina longicruris* (57.8 ± 2.8 wt%) and RO (60.4 ± 5.1 wt%). However, lower contents were reported by Pangestuti et al. [66] for brown seaweeds *Padina australis* (48.98 ± 1.01 wt%) and *Sargassum polycystum* (36.55 ± 1.09 wt%). The carbohydrate content of macroalgae can differ greatly in species from different taxonomic groups or even within species of the same genus [61]. In recent years, brown seaweed carbohydrates have received great interest as valuable biomass sources with biological activities, finding applications in functional foods, pharmaceuticals and cosmeceuticals [67], and for biofuel production [48].

Marine algae are rich sources of polysaccharides [68] with potential use as anti-inflammatory, immune-stimulating, antibacterial, antiviral, antioxidant, and anticancer agents [69]. RO showed a high content of holocellulose (49.2 ± 1.3 wt%) with α-cellulose and hemicellulose contents of 43.4 ± 2.0 wt% and 5.8 ± 0.7 wt%, respectively. The total lignin content of RO was 18.9 ± 2.5 wt% (18.2 ± 2.4 wt% of insoluble lignin and 0.7 ± 0.1 wt% of soluble lignin). Lower contents of holocellulose and total lignin were found in brown seaweed *Padina tetrastromatica* (28.4 ± 0.60 wt% and 3.65 ± 0.07 wt%, respectively) [70]. The high cellulose content found in seaweeds makes them promising sources of fermentable sugars for ethanol production [71]. In addition, marine polysaccharides can be considered potential dietary fiber sources [72].

### 3.2. Fatty Acids Profile

**Figure 2:**
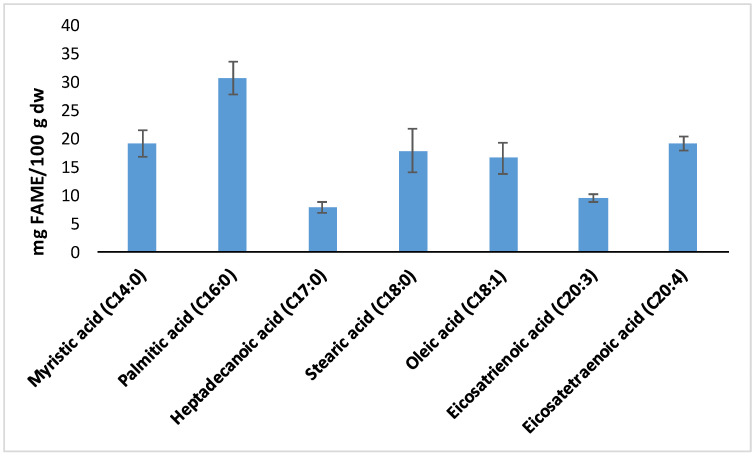
Fatty acids composition obtained for RO (mean ± SD, n = 3).

The fatty acid profile of RO (Figure 2) showed the presence of myristic acid (C14:0), palmitic acid (C16.0), heptadecanoic acid (C17:0), stearic acid (C18:0), oleic acid (C18:1), eicosatrienoic acid (C20:3), and eicosatetraenoic acid (C20:4). According to these results, RO presented a higher content of unsaturated fatty acids (MUFAs and PUFAs) compared to saturated fatty acids (SFAs), with potential use in nutraceutical goods [59]. PUFAs can benefit skin barrier protection and enhance other biological functions, contributing to avoid obesity and regulating inflammatory responses [73]. The main fatty acid present in RO was palmitic acid (30.8 ± 3.0 mg/100 g), followed by myristic acid (19.3 ± 2.4 mg/100 g) and eicosatetraenoic acid (19.2 ± 1.3 mg/100 g). The lowest content was found for heptadecanoic acid (7.9 ± 0.9 mg/100 g). These results are in line with those reported by Cebrián-Lloret et al. [44], who found C16:0 and C14:0 as the main fatty acids present in RO. Similarly, Jayasinghe et al. [74] reported C16:0 as the major fatty acid found in four seaweeds: *Ulva lactuca, Sargussum wightii, Sargussum turbinaria*, and *Kappaphycus alvarezii*. In another work, Dawczynski et al. [75] found trace levels of C17:0 in different seaweed varieties. Myristic and palmitic acids were shown to possess antioxidant and anti-inflammatory properties [44], while palmitic, stearic and oleic acids were reported to be potential feedstocks for biofuels production [76]. ω-3 PUFAs, such as eicosatrienoic acid, are well known for presenting numerous health benefits, such as anticancer and anti-inflammatory properties, and reducing the risk of cardiovascular diseases and diabetes [59,77,78].

### 3.3. Monosaccharides Profile

The monosaccharides composition of RO (Table 2) showed glucose as the main component (13.2 ± 1.0 wt%), derived from cellulose, followed by glucuronic acid (9.3 ± 0.5 wt%) and lower contents of D-galactose (2.6 ± 0.3 wt%), fucose (2.7 ± 0.3 wt%), xylose (1.3 ± 0.2 wt%), cellobiose (0.7 ± 0.1 wt%), and mannose (0.6 ± 0.2 wt%). These results are in line with those found in previous studies for other seaweeds. Rupérez et al. [79] reported a fucose content of 2.47 ± 0.13 wt% in brown seaweed *Fucus vesiculosus* and a mannose content of 0.54 ± 0.09 wt% in *Laminaria digitate*. Rabemanolontsoa and Saka found hemicelluloses mostly composed of xylose, galactose, mannose, and arabinose in relatively equal amounts in different macrophytes growing in Lake Biwa [80]. Ferreira-Anta et al. [20] reported higher contents of fucose (6.38 ± 0.02 wt%) and mannose (1.91 ± 0.05 wt%) in RO, and lower values of glucose (11.69 ± 0.12 wt%), glucuronic acid (1.65 ± 0.01 wt%), and xylose (0.68 ± 0.02 wt%). Steinbruch et al. [81] also found glucose as the major monosaccharide present in *Ulva* sp. Brown seaweeds were reported to contain significant amounts of glucose, being stored in the main carbohydrate reserve substance, laminarin [82]. Glucuronic acid-based polysaccharides are of interest in biomedical [83] and cosmetics [84] sectors. The monosaccharides composition found in RO suggested this seaweed as a potential biomass for the production of carbon-based products, such as biofuels and bioplastics [85,86].

### 3.4. Minerals Composition

Brown macroalgae are rich sources of minerals, with contents ranging 14 to 35 wt% [87]. Their concentration and composition are influenced by geographical location and species, as algae can selectively absorb minerals from the surrounding seawater [63]. Seaweeds were reported to contain more minerals and possess a higher ability (>10^6^) to concentrate rare earth elements compared to terrestrial plants [38] by specifically accumulating minerals from nearby seawater in their thalli [88].

RO showed high amounts of macrominerals (Table 3), with decreasing order concentrations of Ca > K > Na > S > Mg and values of 413.6 ± 3.4 mg/100 g, 259.0 ± 3.6 mg/100 g, 200.3 ± 0.1 mg/100 g, 199.9 ± 3.6 mg/100 g, and 101.9 ± 3.5 mg/100 g, respectively. RO also presented appreciable quantities of trace elements (Mn, Mo, Se, and Cu). A similar trend was reported by Premarathna et al. [89] in brown algae *Gracilaria corticata*. Santoso et al. [90] also found Na, Ca, Mg, and K as major minerals present in Japanese brown alga *Laminaria japonica*, with concentrations of 590 mg/100 g, 75 mg/100 g, 120 mg/100 g, and 42 mg/100 g, respectively, showing higher Na and Mg contents and lower Ca and K values compared to RO. Ca was mainly found in RO, being an essential mineral for human health found in seaweeds at high concentrations [55]. Brown seaweed *Sea pasta* and *Wakame* were reported to contain approximately eight times more calcium than milk, making them excellent Ca sources for children growth, osteoporosis prevention, and pre- and post-menopausal women [25]. Oucif et al. [91] concluded that brown seaweeds have higher K and Na contents compared to green and red algae, with potassium being essential for seaweed growth and metabolic activity; certain seaweeds accumulate K more than other elements [92]. RO showed a Na/K ratio of 0.77, in agreement with previous investigations reporting low Na/K ratios (below 1.5) in brown seaweeds [93]. This is a very interesting result from a nutritional point of view, as foodstuff containing high levels of Na and K were linked to an increased risk of hypertension. For example, Na/K ratios in sausages and olives were reported to be 4.89 and 43.63, respectively [94]. In addition, recent studies have shown seaweed minerals rich extracts as efficient liquid fertilizers on various crops, such as *Triticum aestivum* var. Pusa Gold and wheat [95].

RO showed concerning levels of some toxic metals, such as Ni (0.2 ± 0.1 mg/100 g), Cd (0.002 ± 0.001 mg/100 g), and As (0.3 ± 0.1 mg/100 g), with potentially negative effects on human health [96]. This is an important aspect to be considered for seaweed consumption; however, by now, there is no legislation in the EU addressing limits for toxic elements in edible seaweeds [97]. Maximum levels for heavy metals and metalloids were set by Commission Regulation No. 1881/2006 [98], amended by Regulation No. 629/2008 [99], in a range of foodstuffs including seafood, but seaweeds were not included in the list [100]. In the recent Regulation (EU) 2021/1323 [101], a maximum level of Cd (3 mg/kg) was established in certain foodstuffs, including food supplements made primarily or exclusively from dried seaweed, but without controlling maximum levels of all toxic elements found in seaweeds. In this work, a lower Cd value was obtained in RO compared to the established limit. Brown seaweeds were proposed as biosorbents to remove toxic heavy elements from the environment due to their high metal biosorption capacity [102].

### 3.5. Antioxidant Activity and Total Phenolics Content

Seaweeds can be considered excellent antioxidant sources with great potential for applications in the food, feed, cosmetics, and pharmaceutical industries [36]. Only a few reports can be found comparing antioxidant properties of seaweeds from different groups [103]. Devi et al. found high antioxidant activity and phenolics content for three different Indian brown seaweeds [104]. The antioxidant activity of RO was evaluated using DPPH, ABTS, and FRAP assays in ethanolic extracts obtained by MAE (with a yield of 16.5 ± 1.5 wt%). The assessment of antioxidant properties by different methods enables a better understanding of the mechanisms of the antioxidative action of seaweed extracts [105].

The DPPH (2,2-diphenyl-1-picrylhydrazyl) free radical scavenging activity assay is generally used as an easy, rapid, and useful method to evaluate potential antioxidants and radical scavengers [106]. A value of 3.0 ± 0.4 mg TE/g was obtained for RO by the DPPH test, being much higher than those obtained for green seaweed *Cladophora glomerata* (0.69 ± 0.03 mg TE/g) [107]. The ABTS (2,2′-azino-bis(3-ethylbenzothiazoline-6-sulphonic acid)) assay is a simple method for determining the antioxidant activity of natural products [108], and it is based on antioxidants reaction both by single electron transfer (direct reduction of the ABTS^+^ radical) or radical quenching by hydrogen atom transfer [77]. The ABTS value obtained in RO was 4.5 ± 0.3 mg TE/g. A lower value was reported by Dang et al. [108] in brown algae *Sargassum linearifolium* (2.02 ± 0.51 mg TE/g). The FRAP assay measures the ability of an antioxidant to reduce a ferric oxidant (Fe^3+^) to a ferrous complex (Fe^2+^) by electron transfer, indicating the capacity of the compound to reduce reactive species [109]. A FRAP value of 4.7 ± 0.3 mg TE/g was found for seaweed RO, in agreement with results obtained by Elkhateeb et al. [110] for brown seaweed *sea spaghetti* (4.7 mg TE/g).

Ak and Turker [111] found greater antioxidant activity for brown seaweeds *Scytosiphon lomentari* and *Cystoseira barbata* compared to green and red seaweeds. Similarly, El-Manawy et al. [61] concluded that higher amounts of polyphenols and DPPH radical scavenging activity can be found in brown seaweeds compared to red and green species. The antioxidant activity of extracts depends on the presence of small molecular compounds, such as peptides, polysaccharides, and phenolic compounds [109]. Seaweeds can produce different antioxidant types to counteract environmental stresses [112]. The considerable antioxidant activity found for RO highlights its great potential as plant-based source of antioxidants and phenolic compounds for applications in several sectors, including food, pharmaceutical, and cosmetic industries [44].

RO showed a total phenolics content (TPC) of 2.7 ± 0.2 mg GAE/g, being much higher compared to other seaweeds from the same species, such as *Ishige okamurae* (0.63 mg GAE/g) [113]. Zhang et al. [114] reported TPC values ranging 0.29 ± 0.01 to 1.42 ± 0.01 mg GAE/g for brown algae, presenting RO higher TPC. Phenolic compounds in marine algae are usually well correlated with antioxidant activity, depending on the solvent and algal species. Essentially, an increase in TPC values may also increase antioxidant properties [115]. Spearman’s correlation was used to relate TPC and antioxidant activity (DPPH, ABTS and FRAP) of RO extracts. TPC showed a strong positive correlation with DPPH and FRAP assays (*r* = 1.000), showing similar predictive capacity of antioxidant activity for RO. These results suggested that phenolic compounds were the main substances contributing to the antioxidant capacity of RO extracts. However, ABTS did not present significant correlations with TPC, DPPH, and FRAP variables (*p* > 0.05). The positive correlation between TPC and DPPH antioxidant activity is well documented in brown and red algal extracts [116]. TPC values can vary between species, presenting green seaweeds with higher free radical scavenging properties, followed by brown seaweeds and red algae [117]. These variations in TPC and antioxidant activity among different seaweed species can be attributed to external environmental factors, such as salinity levels, collection depth, nutrients, light intensity, and seasonal impacts, and several inherent factors, such as length and age [118]. Phenolic compounds are commonly found in marine seaweed extracts, showing several biological activities, including antioxidant properties [104]. Seaweeds also contain other active compounds, such as polysaccharides, pigments, proteins, or peptides, which could also contribute to antioxidant activity [119].

### 3.6. Phenolics Profile

**Figure 3:**
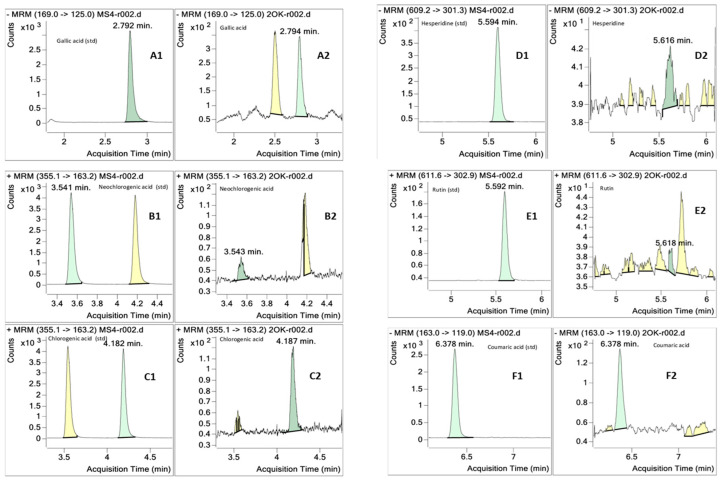
LC-MS/MS total ion chromatograms (TICs) of standards (1) and extracted compounds (2) in RO. (**A1**,**A2**): gallic acid, (**B1**,**B2**): neochlorogenic acid, (**C1**,**C2**): chlorogenic acid, (**D1**,**D2**): hesperidin, (**E1**,**E2**): rutin, (**F1**,**F2**): coumaric acid.

An HPLC-ESI-MS/MS analysis using the multiple reaction monitoring mode (MRM) was carried out to identify phenolic compounds present in RO seaweed. Both negative and positive ionization modes were considered to optimize polyphenols identification. The main parameters used (retention time (RT), coefficient of determination (R^2^), and concentration of polyphenols present in RO) are presented in Table 4, whereas the obtained total ion chromatograms are shown in Figure 3. Six main polyphenols were detected in RO extracts: gallic acid, chlorogenic acid, neochlorogenic acid, rutin, hesperidin, and coumaric acid, in agreement with the reported results in other brown seaweeds [120].

Gallic acid was identified at 2.79 min (Figure 3(A1,A2)) and a concentration of 20.7 ± 1.5 mg/g, showing a molecular ion [M-H]^−^ at *m*/*z* 169 and a characteristic ion at *m*/*z* 125 ([M-H−44]^−^), corresponding to the loss of CO_2_ molecule. Gallic acid was the main phenolic present in RO, being known as an abundant and potent antioxidant found in brown algae extracts [121]. Neochlorogenic acid (Figure 3(B1,B2)) and chlorogenic acid (Figure 3(C1, C2)) were identified at 3.55 min and 4.19 min, respectively, both showing [M+H]^+^ ions at *m*/*z* 355.1 [122], with concentrations of 2.2 ± 0.1 mg/g and 4.9 ± 0.2 mg/g, respectively. Hesperidin (Figure 3(D1,D2)) and rutin (Figure 3(E1,E2)) isomers were detected around 5.6 min, with concentrations of 1.5 ± 0.4 mg/g and 0.6 ± 0.9 mg/g and [M-H]^−^ ion at *m*/*z* 609.2 and [M+H]^+^ ion at *m*/*z* 611.6, respectively. Finally, coumaric acid (Figure 3(F1,F2)) was found at 6.37 min and a concentration of 1.4 ± 0.9 mg/g, similar to hesperidin, with an [M-H]^−^ ion at *m*/*z* 164.

### 3.7. Thermal Stability

**Figure 4:**
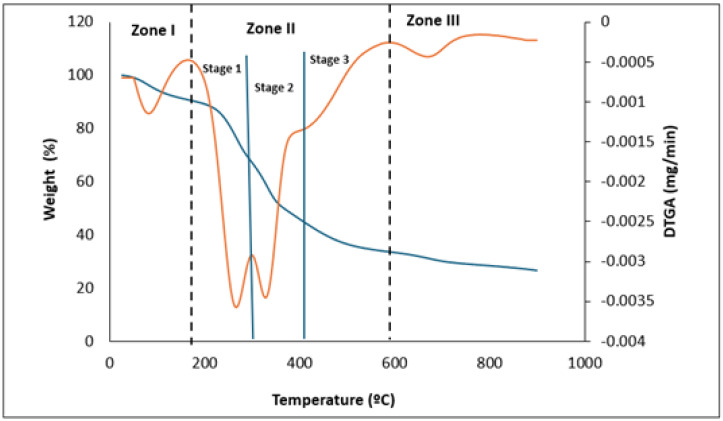
TGA (blue) and DTGA (orange) curves obtained for RO showing different decomposition stages.

TGA and DTG curves (Figure 4) obtained for RO, under a nitrogen atmosphere, showed three different decomposition zones, including dehydration (I), maximum degradation (II), and the decomposition of carbonaceous solids (III). Similar results were reported by Ross et al. [123] for brown algae *Macrocystis pyrifera*. The first zone (I, 52–180 °C) was associated with the removal of moisture and the loss of low-molecular-weight volatile compounds. The second degradation zone (II, 180–600 °C) was related to the decomposition of different biopolymer fractions [124]. Three different decomposition stages were observed in this zone: the decomposition of hemicellulose and initial degradation of cellulose (stage 1, 150–268 °C); lignin degradation and final degradation of cellulose (stage 2, 268–409 °C); and final degradation of lignin (stage 3, above 409 °C) [125]. Mohammed et al. [96] reported that the main degradation of seaweeds ranged between 190 and 390 °C, with the maximum weight loss at 260 °C, showing carbohydrate and protein decomposition at 180–270 °C and 320–450 °C, respectively. Finally, in the third zone (III, 600–800 °C), a minor weight loss was observed, which was associated with the further decomposition of the remaining solid residue (i.e., char carbonization) [124].

## 4. Conclusions

This study highlights the potential of brown seaweed *Rugulopteryx okamurae* (RO) as a rich natural source of bioactive compounds with antioxidant activity to be used in different applications, providing functional and nutritional beneficial effects. A full characterization of RO collected from the Gibraltar coast was performed for the first time, showing appreciable levels of carbohydrates, fats, and ashes. The main fatty acid present in RO was palmitic acid (C16:0, ca. 31 mg/100 g), followed by similar contents (ca. 19 mg/100 g) of myristic acid (C14:0) and eicosatetraenoic acid (C20:4). Holocellulose was the most abundant polysaccharide fraction, with ca. 43 wt% of cellulose. High levels of glucose and glucuronic acid were found, along with macrominerals (Ca > K > Na > S > Mg). RO also presented some levels of toxic elements, such as Ni, Cd, and As, which should be considered before human consumption. RO showed significant TPC and polyphenol contents with potential antioxidant properties, with gallic acid being the most abundant phenolic compound (ca. 21 mg/g). In conclusion, seaweed RO presents great potential as a functional food supplement in the food and feed industries for the development of new ingredients with nutritional value. Moreover, the high mineral content and ability of RO to accumulate toxic metals from seawater open new insights for using this brown alga as an absorbent material or biomarker in ecological investigations. These valorization approaches will contribute to reducing the environmental problems associated with this invasive species accumulation by creating new functional products and considering the principles circular economy.

## Figures and Tables

**Table 2:** Monosaccharides present in RO (mean ± SD, n = 3).

Monosaccharide	This Study (wt%, dw)	Other Studies (wt%, dw) [20]
Fucose	2.7 ± 0.3	6.38 ± 0.02
D-galactose	2.6 ± 0.3	2.76 ± 0.07
Glucose	13.2 ± 1.0	11.69 ± 0.12
Xylose	1.3 ± 0.2	0.68 ± 0.02
Mannose	0.6 ± 0.2	1.91 ± 0.05
Cellobiose	0.7 ± 0.1	−
Glucuronic acid	9.3 ± 0.5	1.65 ± 0.01

**Table 3:** Minerals content of RO (mean ± SD, n = 3).

Element	mg/100 g dw	Element	mg/100 g dw
Ca	413.6 ± 3.4	Cu	0.2 ± 0.1
K	259.0 ± 3.6	Mn	0.2 ± 0.1
Na	200.3 ± 0.1	Rb	0.2 ± 0.1
S	199.9 ± 3.6	Ni	0.2 ± 0.1
Mg	101.9 ± 3.5	Ti	0.03 ± 0.01
Sr	23.1 ± 0.7	V	0.02 ± 0.01
P	16.2 ± 0.9	Pb	0.02 ± 0.01
Fe	6.0 ± 0.6	Cr	0.02 ± 0.01
Si	2.0 ± 0.2	Mo	0.02 ± 0.01
Al	1.1 ± 0.2	Se	0.02 ± 0.01
Zn	0.6 ± 0.2	Co	0.02 ± 0.01
As	0.3 ± 0.1	Ag	0.002 ± 0.001
Sn	0.3 ± 0.1	Cd	0.002 ± 0.001

**Table 4:** Polyphenols analysis by HPLC–MS/MS in RO (mean ± SD, n = 3).

Analyte	RT (Min)	R^2^	Precursor Ion (*m*/*z*)	Product Ion (*m*/*z*)	Ionization Mode	Collision Energy (V)	mg/g dw
Gallic acid	2.79	0.999	170	125, 51	ESI (−)	14, 14, 40	20.7 ± 1.5
Neochlorogenic acid	3.55	0.999	354	163.2	ESI (+)	10, 40, 40	2.2 ± 0.1
Chlorogenic acid	4.19	0.999	354	163.2	ESI (+)	10, 40, 40	4.9 ± 0.2
Rutin	5.57	0.999	610	465.2, 302.9	ESI (+)	10, 10	0.6 ± 0.9
Hesperidin	5.58	0.999	610	301.3	ESI (−)	28, 40	1.5 ± 0.4
Coumaric acid	6.37	0.999	164	119, 92.9	ESI (−)	16, 30, 40	1.4 ± 0.9

## Data Availability

Data is contained within the article.
